# Real-World Study of Definisse Threads for Facial Reshaping in Indian Patients: REDEFINE FACE Study

**DOI:** 10.7759/cureus.58258

**Published:** 2024-04-14

**Authors:** Sukhbir Singh, Nitin Sethi, Malavika Kohli, V S Rathore, Indu Ballani, Madhuri Agarwal, Rickson Pereira, Shefali Trasi Nerurkar, Isha Kaushik, Sanjana Dadhich, Chetan Y Patil

**Affiliations:** 1 Plastic Surgery, Resplendent Cosmetic Studio, New Delhi, IND; 2 Plastic Surgery, Fortis Hospital, Ludhiana, IND; 3 Dermatology, Skin Secrets, Jaslok Hospital & Research Centre, Mumbai, IND; 4 Plastic Surgery, Kaayakalp Clinic, Kolkata, IND; 5 Dermatology, BLK-Max Super Speciality Hospital, New Delhi, IND; 6 Dermatology, Yavana Aesthetic Clinic, Mumbai, IND; 7 Dermatology, Dr Rickson's Dermatherapie Clinic, Mumbai, IND; 8 Dermatology, Dr. Trasi's La Piel Skin Clinic, Mumbai, IND; 9 Medical Affairs, A. Menarini India Pvt Ltd, Delhi, IND; 10 Medical Affairs, A. Menarini India Pvt Ltd, Mumbai, IND

**Keywords:** face lift, thread lifting, facial thread-lifting, thread lift, definisse threads

## Abstract

Introduction: In recent years, thread lifting has gained popularity as a less invasive cosmetic surgery. It helps raise and realign sagging tissue. The newest thread type for thread lifting procedure is poly-lactic acid-polycaprolactone (PLCL) Definisse threads (RELIFE S.r.l., Florence, Italy). These are fourth-generation absorbable suspended barbed threads. Their double action involves an immediate elevating impact through mechanical action and, over time, the promotion of histological rejuvenating activity through inducing fibroblasts and the synthesis of elastin, hyaluronic acid, and collagen.

Objectives: The REDEFINE FACE study assessed the effectiveness and tolerance of Definisse threads in a real-world setting for facial reshaping in patients in India.

Methods: This multicenter, retrospective observational research included patients treated with Definisse threads for face contouring.

Results: Three hundred seventeen patients with a mean follow-up of 4.9 months and an average age of 48.6 years participated in the study. All patients underwent thread lifts using either a single or a combination of Definisse thread reshaping techniques. The Global Aesthetic Improvement Scale for Physicians and Subjects (PGAIS and SGAIS) reported improvement instantly following the treatment (mean score- 3.23 and 3.18, respectively). Improvements continued to enhance during the follow-up visits (mean score- 4.09 and 4.03, respectively). Following the procedure, 96% of patients on the PGAIS and SGAIS exhibited enhancement. Most procedure-related side effects were minor and went away on their own in a few days without the need for proactive care.

Conclusion: The results of this real-world analysis showed that the latest Definisse threads effectively achieve facial reshaping on patients in India and have both immediate and long-term effects. Treatment was generally well tolerated, and no patient experienced serious adverse events.

## Introduction

Aging is a natural process, and aging of the face is especially progressive and affects facial tissues, such as skin, bones, ligaments, muscles, and fat tissue [1-3]. Aesthetic medicine, more substantially face aesthetics, has made significant progress over the past decade, leading to a change from more invasive procedures to minimally invasive procedures [2]. Thread lifting is one of the aesthetic procedures that has developed significantly in recent years. Today, the most sought-after aesthetic procedure is the minimally invasive thread lift technique, which lifts and aligns the sagging tissue. This newer, less invasive technique shortens recovery time and reduces the risk of complications with modest aesthetic improvements [2,3]. Subcutaneous placement of these threads can enhance the definition of facial features, tighten the tissue, and realign the superficial fat pads [4,5]. Thread lift techniques can be done alone or in conjunction with other face rejuvenation treatments, including neurotoxins, hyaluronic acid fillers, and lasers [5-8]. Absorbable polymers having a defined lifespan, such as polydioxanone (PDO), polyglycolic acid, poly-L-lactic acid (PLLA or PLL), and poly-lactic acid-polycaprolactone (P(LA-CLA) or PCLA or PLCL), are frequently used to make threads for cosmetic operations [5,9,10].

Definisse threads (RELIFE S.r.l., Florence, Italy) are the latest 4th generation absorbable threads. These are synthetic, monofilament barbed threads with convergent, bidirectional barbs [11-13]. They are ε-caprolactone and L-lactide co-polymers. While ε-caprolactone is a semicrystalline substance with rubbery qualities, L-lactic acid is a crystalline and hard substance. The copolymer poly (ε-caprolactone-co-L-lactic acid) of threads presents a viable substitute for PDO or PLLA threads because of its convenient degradation profile and well-established biocompatibility [5,11,12]. These threads can safely realign facial tissue with long-lasting results by stabilizing and elevating drooping face tissue. Patients experience instant results and satisfaction from this thread procedure because it is tolerated very well, the patient recovers more quickly, and the procedure does not take longer to complete [11,12].

Definisse threads were introduced in India in early 2021. Since then, they have gained popularity due to their dual method of action, capacity to lift and realign sagging facial tissue, longer-lasting effects, and faster recovery times [13]. Two types of Definisse thread are available in India: double-needle thread 12 cm or 23 cm depending upon barb thread length and free-floating thread 12 cm without any needle [12,13]. Therefore, this multicenter retrospective REDEFINE FACE study was designed and conducted with the objective of assessing the real-world effectiveness and tolerance of the Definisse threads when used in Indian patients undergoing face contouring.

## Materials and methods

This study was a retrospective, multicenter, observational conducted in a real-world setting. The analysis in this study was limited to patients who received face contouring treatment between September 2021 and December 2023 utilizing Definisse threads (double needle 12 cm/23 cm or free-floating threads) and completed at least one follow-up post-procedure. Patient demographics, including age and gender, were documented, as were treatment specifics, including the type and quantity of threads used, the method utilized, any adverse effects, and the therapy's results as measured by the Physician and Subject Global Aesthetic Improvement scale (PGAIS and SGAIS). GAIS was assessed as score 1: grade 'worse' (appearance is worse than the original condition); score 2: grade 'no change' (appearance is essentially same as the original condition); score 3: grade 'improved' (obvious improvement in appearance than original condition but touch up is required); score 4: grade 'much improved' (marked improvement in appearance than original condition but not completely optimal); score 5: grade 'very much improved' (optimal aesthetic improvement) [13].

Unreal patient expectations, noticeable personality disorders, prior permanent filler treatment in the procedure area, anticoagulant treatment, bleeding disorders, drug abuse, hypersensitivity to lidocaine, adrenaline, ε-caprolactone and/or L-lactide, immunologically compromised diseases, local or systemic infection, any other local skin disease, uncontrolled diabetes, pregnancy, and nursing females are all considered contraindications to the thread lift procedure [13].

A complete patient assessment was carried out to determine the best suitable reshaping techniques using Definisse threads, and accordingly, patients were marked for reshaping techniques [5,14]. The procedure was carried out after injecting the local anesthetic, 2% lignocaine, and 1:1,00,000 adrenaline per side of the face (6-7 ml total) [5]. Based on individual patient assessment, thread reshaping techniques were performed either alone or in combination with two techniques [5,14]. The treated region was covered with anti-infective ointment for a week following the treatment. For the first four weeks, all patients were recommended to avoid dental operations, sauna visits, strenuous exercise, and face massages to prevent complications like thread migration or protrusion. Additionally, they were instructed not to use force when washing and drying the treated area. They were also asked to carefully apply makeup if needed [5,13].

SPSS Statistics version 10.0 was used for data analysis. The results were noted in the form of descriptive statistics. Frequency and percentages were used to analyze categorical data, while mean and standard deviation was used for summarizing continuous data. Parametric paired data were analyzed using a two-tailed paired t-test, and a p-value < 0.05 was considered statistically significant for all comparisons.

## Results

**Figure 1:**
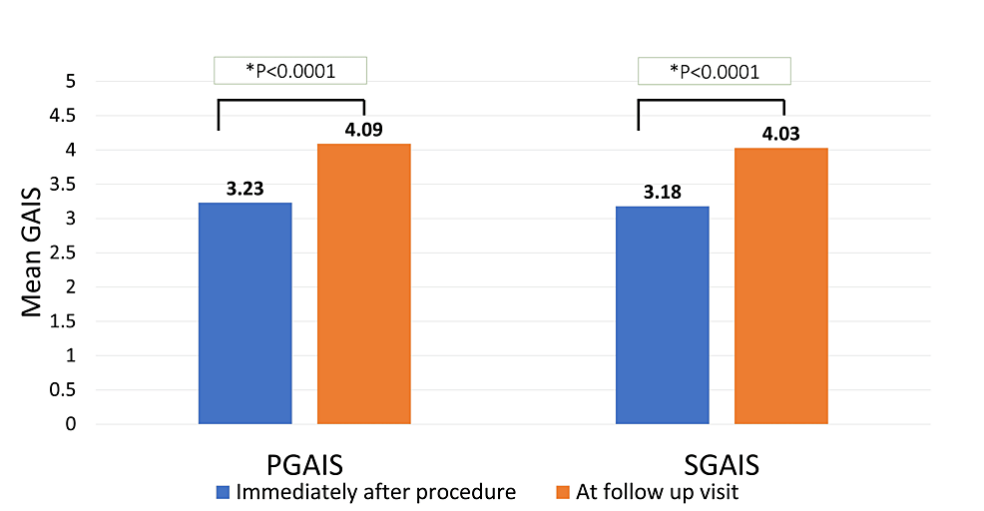
Mean GAIS rating immediately after the procedure and at the follow-up visit * p-value at the follow-up visit compared to immediately after the procedure by two-tailed paired t-test, p < 0.05- significant; GAIS: Global Aesthetic Improvement scale; PGAIS: Physician Global Aesthetic Improvement scale; SGAIS: Subject Global Aesthetic Improvement scale

Three hundred and seventeen patients from 8 centers with ages ranging from 23 to 81 years and a mean of 48.56±10.16 years were considered for analysis. Patients came for follow-ups from 1 to 24 months, with an average visit time of 4.9 months. Most of the patients who participated in the study were females (84.2%), while 15.8% were male. The specifics of various types of techniques performed in the study (Table 1). The mean scores for the PGAIS and SGAIS improved soon after the treatment (3.23±0.59 and 3.18±0.52, respectively), and they continued to improve significantly during the subsequent visits (4.09±0.73 and 4.03±0.81 respectively, p-value <0.0001 for both parameters at follow up visit vs. immediately after procedure) (Figure 1).

**Table 1:** Details of techniques performed using Definisse threads n: number of patients; JR: Jawline reshaping; MR: Malar reshaping; ERT: Eyebrow reshaping technique; ORV: Oval reshaping-V; ORH: Oval reshaping-H; STR: Soft tissue repositioning (STR); LR: Lateral reshaping

Facial reshaping technique	Objective	Type of threads used	n	Percentage of patients
JR	For reshaping of the frame of the jawline and repositioning ptotic tissues of the lower face, so as to Increase the width and definition of mandibular angles, which is not preferred by many Asians	Double-needle 12CM	67	21.1
MR	For reshaping the facial frame by lifting the superficial fat compartments of the malar and cheekbone area superiorly and laterally, so as to improve nasolabial fold & marionette line depending on the Individual patients and thread placement		25	7.9
JR+MR	Combination of two techniques to increase definition of mandibular angles and also to improve nasolabial fold &/or marionette line		74	23.3
ERT	To enhance the arch of the eyebrows		1	0.3
ORV	For reshaping the facial frame in order to make the face appear oval by lifting the mid- and lower-face superficial fat compartments of the cheek		64	20.2
ORH	For vertically reshaping the frame of the face & is generally performed in patients with large and round face		5	1.6
STR	Reposition the tissue of the malar area, remodeling the treated area, in patients affected by mild to moderate ptosis	Free-floating 12CM	23	7.3
JR+STR	Combination of JR and STR techniques to increase the definition of mandibular angles & to reposition the tissue of the malar area	Double-needle 12CM and free-floating 12CM	21	6.6
ORV+STR	For reshaping the facial frame to make the face appear oval & to reposition the tissue of the malar area		2	0.6
LR	For reshaping the facial frame by lifting the superficial fat compartments of a large part, that is, the upper and lower areas, of the cheek superiorly and laterally	Double-needle 23CM	33	10.4
Neck reshaping (W shape technique)	To reposition the mild to moderate sagging neck tissues to reduce laxity and wrinkles		2	0.6

**Figure 2:**
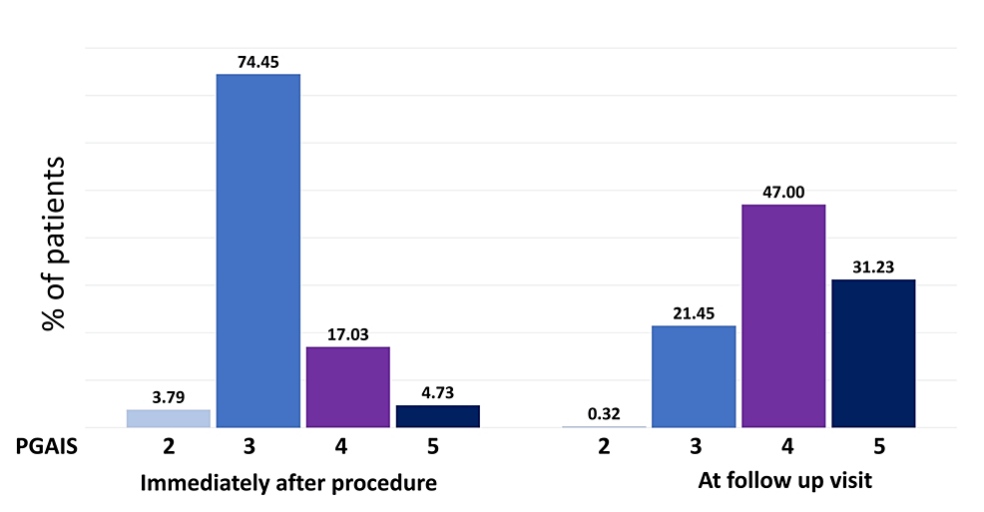
Percentage of patients showing improvement on PGAIS rating immediately after the procedure and at the follow-up visit PGAIS: Physician Global Aesthetic Improvement scale

**Figure 3:**
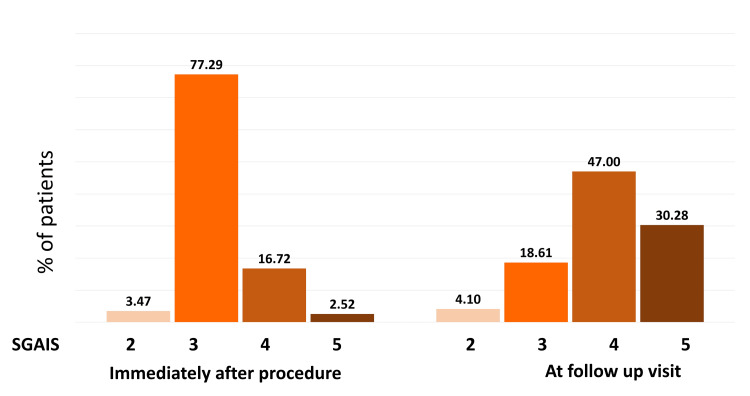
Percentage of patients showing improvement on SGAIS rating immediately after the procedure and at the follow-up visit SGAIS: Subject Global Aesthetic Improvement scale

**Figure 4:**
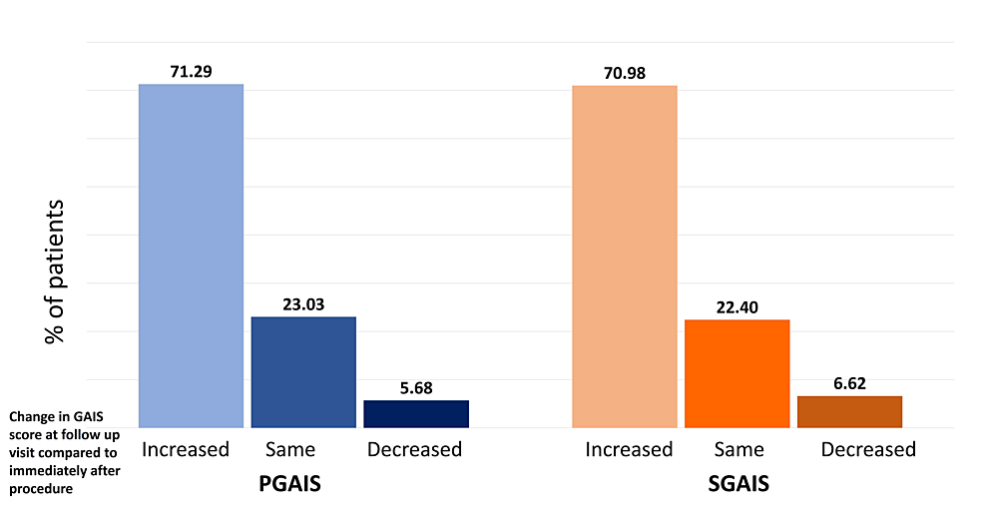
Change in GAIS score at follow-up visit compared to immediately after the procedure GAIS: Global Aesthetic Improvement Scale; PGAIS: Physician Global Aesthetic Improvement Scale; SGAIS: Subject Global Aesthetic Improvement Scale

Furthermore, the majority of patients reported immediate treatment outcomes as “three, that is, appearance improved compared to pretreatment” in PGAIS (74.45%) and SGAIS (77.29%). At the follow-up appointment, 47% of patients reported the treatment outcome as "four, marked improvement in appearance” compared to pretreatment in PGAIS and SGAIS (Figures 2-3). Overall, 96% of patients exhibited improvement right after the treatment. Subsequent analysis revealed that about 71% of patients had a one-point increase in their GAIS score at the follow-up appointment compared to immediate post-procedure (Figure 4). Furthermore, about 23% of patients reported the same GAIS score at follow-up visits compared to post-procedure. Only about 6% of patients showed a decrease in GAIS score (either from 5 to 4 or 4 to 3 or 3 to 2); however, none showed worsening condition (GAIS score 1) at follow-up.

**Figure 5:**
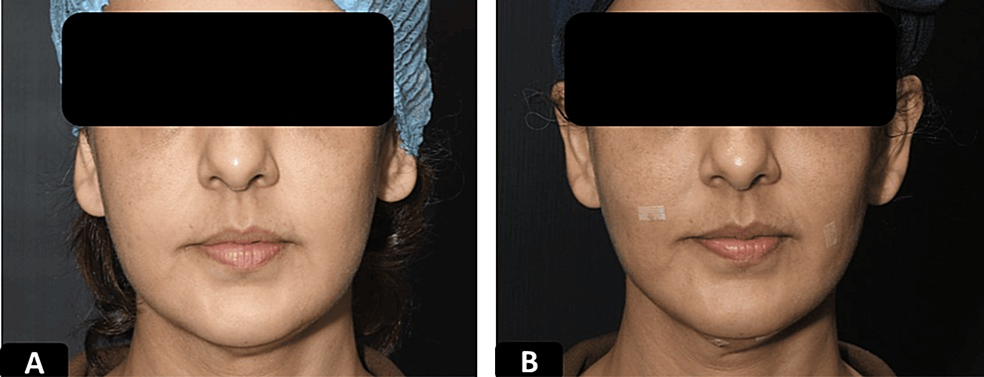
Clinical photographs of a patient who underwent LR procedure A 29-year-old female underwent an LR (Lateral reshaping) procedure using 1 pair of 23 cm double needle threads. A) Pre-procedure and B) immediately after the procedure, which shows reshaping of the mid and lower face.

**Figure 6:**
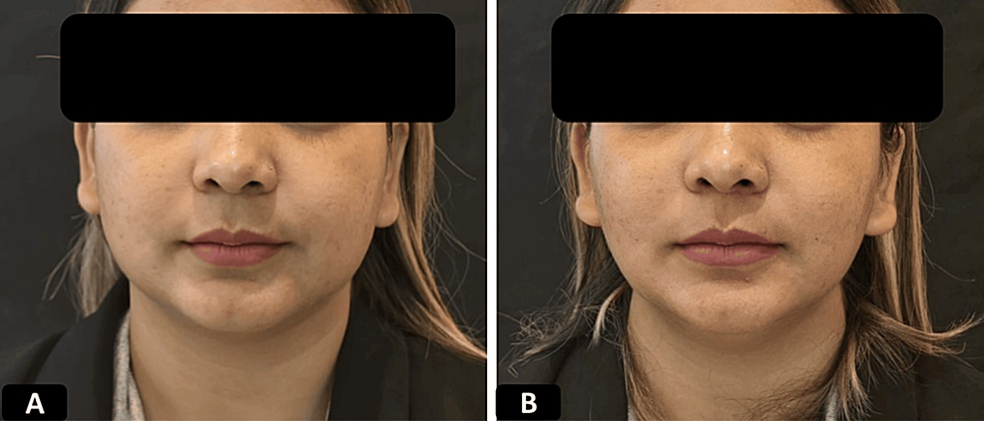
Clinical photographs of a patient who underwent ORV procedure 28 years old female who underwent oval reshaping-V (ORV) procedure using 1 pair of 12 cm double needle threads A) Pre-procedure B) immediately after the procedure showing significant improvement in the shape of mid & lower face

**Figure 7:**
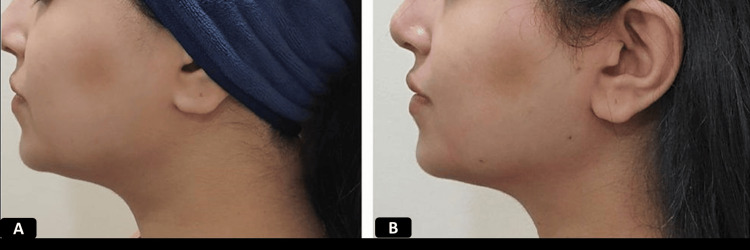
Clinical photographs of a patient who underwent JR procedure 38 years old female who underwent Jawline reshaping (JR) procedure using 1 pair of 12 cm double needle threads A) Pre-procedure B) immediately after the procedure showing more defined jawline

**Figure 8:**
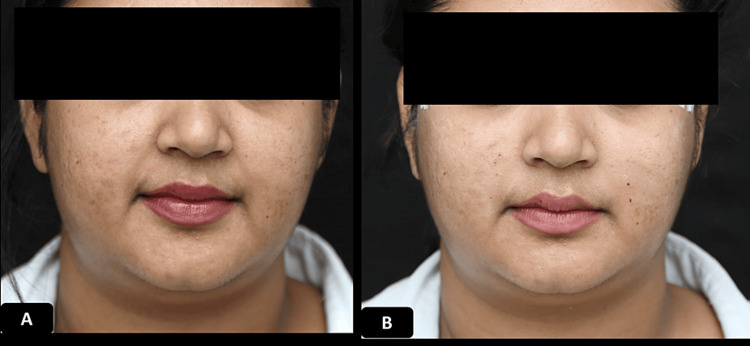
Clinical photographs of a patient who underwent ORV and STR procedure 32 years old female who underwent oval reshaping-V (ORV) and soft tissue repositioning (STR) procedure using 1 pair of 12 cm double needle threads and 2 pairs of 12 cm free floating threads A) Pre-procedure B) immediately after the procedure showing improvement of bilateral nasolabial folds.

The thread implantation procedure is usually associated with some adverse reactions related to the procedure. The current study found that operative site pain/discomfort was the most common adverse reaction, reported by 65% of the study population. Other common adverse reactions noted were bruising (40%), swelling (34%), puckering (21%), nodule development (5%) and thread projection (6%). Most of them were minor and went away on their own on some days without needing proactive care. There was no detectable difference in occurrences of complication with respect to thread techniques. Excision of the protruding thread was done for thread protrusion, which happened during the first week of the treatment. Antibiotics were prescribed to patients who developed nodules for seven days, after which the nodules disappeared. The clinical photographs of patients before and after the thread treatment (Figures 5-8).

## Discussion

Noteworthy progress has been made in facial rejuvenation treatments in recent years, and patients are choosing newer, less invasive procedures more frequently due to their acceptable cosmetic benefits, shorter downtimes, and increased safety. Recently, minimally invasive procedures, specifically thread lifts, have also gained popularity in India because of their capacity to realign soft tissues while ensuring a faster recovery [2,13].

Definisse threads provide a lifting effect that supports and realigns face tissue when inserted subcutaneously because of their mechanical and bio-stimulatory mechanism. Its histologically renewing activity gradually induces fibroblasts and the production of elastin, hyaluronic acid, and collagen around the thread, which is stable even after the thread has been reabsorbed for over a year [2,13]. Furthermore, during resorption, the completely degradable thread polymers hydrolyze into tiny molecules, which promotes the synthesis of collagen and hyaluronic acid even more. The expected total time of thread to remain in the tissue is 12 to 15 months, with a lifting action lasting up to three years [3,12,13].

REDEFINE JAW study conducted by Singh et al. analyzed 50 Indian patients who had undergone JR and MR technique using the Definisse 12 CM double needle threads. Following the thread lift, the study found a mean improvement in PGAIS and SGAIS (Min 0-Max 4) with mean scores of 2.82 and 2.7, respectively. At the follow-up, there was even more improvement (PGAIS-3.72 and SGAIS-3.58). Furthermore, the majority of patients assessed their PGAIS (78%) and SGAIS (66%) improvements as "much improved" in comparison to their pre-treatment appearances, just after the treatment. Moreover, 76% of patients on PGAIS and 66% of the patients on SGAIS reported that their looks had "improved very much" during their follow-up appointments. This study showed that Definisse thread-lift gives apparent results in tissue repositioning of Indian patients [13].

Savoia and colleagues assessed the results of thread lift in the areas of the eyebrow, mid-facial region, mandibular area, zygomatic area, and neck using suspension barbed PCLA threads. Of the 37 patients, about 90% considered the facial contouring with thread treatments satisfactory, with 65% describing the results as "excellent”. Furthermore, the ideal aesthetic results were appreciable even after six months of therapy. A decisive face-reshaping action combined with cutaneous response was shown by histopathological investigation of a subset of patients along the thread's length [11].

Another prospective, single-blind research was carried out by Rungsima et al. in Thailand on 27 patients who had facial tissue sagging for 12 months. Definisse 12 or 23-cm double needle threads were used to reshape the mandibular angle. Face sagging showed a clinical improvement as soon as the threads were implanted. At practically all follow-up visits, there was a considerable volume improvement in the submental region, nasolabial folds, and jawline (p value-0.007). Also, most participants (52%) stated a very good lifting result at the earliest of one week. Although most patients could sustain the lifting benefit for up to six months after the treatment, 72% of individuals could do so for up to twelve months [3].

Our real-world, retrospective study showed comparable results in a large study population that was earlier reported by Singh et al., Savoia et al., and Rungsima et al. In our study, facial contouring using Definisse PLCL barbed threads showed improvement in around 96% of patients instantly after the treatment. The instantaneous lifting characteristic of these threads is the primary mechanism for these results. Approximately 71% of the participants in this analysis also reported further improvement in both GAISs at the follow-up visits, and overall, there was statistically significant improvement at follow-up visits compared to immediate post-procedure. This is thought to be caused by the Definisse threads' delayed activity of promoting the synthesis of new blood vessels, elastin, collagen, and hyaluronic acid [11-13].

Adverse reactions recorded in this study were also similar to those previously documented by PLCL thread lift studies [3,11,13]. The barb suspension thread method is typically well-tolerated and involves minimum invasiveness [13-15]. Several variables, including the surgical field, the patient, and the operator, also influence the likelihood of unfavorable results. Furthermore, most unfavorable events may be prevented with cautious patient selection, a well-thought-out treatment, a suitable aseptic environment throughout the treatment, and close attention to certain anatomical features [12-15].

The current study is the largest real-world study conducted in patients using Definisse threads. However, it also has a few constraints, like being retrospective in nature, which may require additional validation in a bigger prospective trial with longer-term follow-ups.

## Conclusions

This real-world analysis showed that Indian patients who have undergone facial reshaping using the newest Definisse threads have an immediate facial reshaping effect. It also showed a significant long-term effect on overall aesthetic improvement. The study also showed that different reshaping techniques are required for the different patients based on their desire and assessment to achieve the best possible results. Overall, the procedure was well tolerated, and no patients experienced life-threatening side effects. Most adverse reactions are resolved on their own or with very limited medical intervention. Therefore, Definisse thread lifting is a minimally invasive effective treatment and can be a good alternative to more invasive procedures like surgical facelifts.
